# Colonization of three *Sphagneticola* species by *Funneliformis mosseae* under cadmium stress is beneficial to phosphatase activity and nutrient uptake in rhizosphere soil

**DOI:** 10.1186/s40529-026-00494-2

**Published:** 2026-05-11

**Authors:** ZhengChao Yu, RuiRui Lu, QiLei Zhang, YuanXiao Jing, ChangLian Peng

**Affiliations:** 1 School of Life Sciences and Food Engineering, Hanshan Normal University, Chaozhou, 521041 China; 2 Guangdong Provincial Key Laboratory of Biotechnology for Plant Development, Guangzhou Key Laboratory of Subtropical Biodiversity and Biomonitoring, School of Life Sciences, South China Normal University, Guangzhou, 510631 PR China; 3 Research Institute of Tropical Forestry, Chinese Academy of Forestry, Guangzhou, 510520 China; 4 Shenzhen Branch, Guangdong Laboratory of Lingnan Modern Agriculture, Key Laboratory of Synthetic Biology, Ministry of Agriculture and Rural Affairs, Agricultural Genomics Institute at Shenzhen, Chinese Academy of Agricultural Sciences, Shenzhen, 518120 China

**Keywords:** Phytoremediation, Phosphatase activity, *Funneliformis mosseae*, Cadmium stress, Rhizosphere regulation, *Sphagneticola*

## Abstract

**Background:**

Soil cadmium (Cd) contamination poses threats to ecosystems and human health, and the plant arbuscular mycorrhizal fungi (AMF) symbiotic system represents a promising green remediation strategy. However, the underlying mechanisms are complex and context-dependent, and systematic comparative studies remain scarce regarding differential regulation of Cd tolerance enhancement in plants of the same genus but different ecotypes. This study used a native species (*Sphagneticola calendulacea*), invasive species (*Sphagneticola trilobata*), and their hybrid as materials. It conducted an integrated analysis of the synergistic effects of inoculating *Funneliformis mosseae* (FM) on the rhizosphere microenvironment and mineral element uptake of three plants under a Cd stress gradient. The objective was to elucidate the interactive mechanisms by which FM enhances plant Cd tolerance and to evaluate its remediation potential.

**Results:**

Results indicate that FM regulation of rhizosphere pH exhibits species specificity but generally alleviates Cd induced acidification. FM significantly enhances acid phosphatase activity in rhizosphere soil and substantially promotes plant phosphorus (P) uptake. FM comprehensively altered plant mineral element uptake, including promoting root accumulation of sodium (Na), magnesium (Mg), and calcium (Ca), shoot potassium (K) allocation, and copper (Cu) and zinc (Zn) absorption.

**Conclusions:**

This study elucidates how FM enhances P uptake and systematically optimizes elemental absorption homeostasis by regulating rhizosphere pH and phosphatase activity. These synergistic effects improve Cd tolerance in Sphagneticola species and highlight the broad potential of AMF-plant symbioses for Cd remediation. It provides crucial theoretical foundations and germplasm selection references for targeted soil restoration using ecologically distinct Sphagneticola ecotypes and their optimal FM partners.

## Background

Soil heavy metal contamination, particularly cadmium (Cd) pollution, has become a global environmental and public health issue due to its high toxicity, strong mobility, and persistence (Liu et al. 2018). Cd accumulates through the food chain, posing a serious threat to ecosystem safety and human health (Chmielowska-Bake et al. 2018; Hu et al. 2019). Phytoremediation techniques, particularly those utilizing hyperaccumulator or tolerant plants to immobilize, extract, or stabilize Cd in soil, are regarded as an economical, environmentally friendly, and sustainable remediation strategy (Cameselle et al. 2019; Ahmad et al. 2021). Plants belonging to the *Sphagneticola* in the Asteraceae family demonstrate potential application value in the ecological restoration of heavy metal-contaminated soils due to their rapid growth, high biomass, and strong adaptability (Zhang et al. 2020). Our research team previously found that hybrids derived from crosses between *Sphagneticola trilobata* and *Sphagneticola calendulacea* can tolerate high Cd environments (Gao et al. 2022). Notably, significant variations in physiological and ecological responses to heavy metal stress, as well as remediation efficiency, is often observed among different species within the same genus. This diversity provides a valuable germplasm pool for screening and developing highly efficient phytoremediation plants (Zhang et al. 2020). However, plant tolerance to heavy metals and remediation efficiency depend not only on genetic traits but are also strongly influenced by complex interactions within the rhizosphere microenvironment (Jing et al. 2014). The rhizosphere serves as a dynamic interface where plants, soil, and microorganisms interact, and its physicochemical properties and biochemical processes directly influence the bioavailability of heavy metals and their uptake and transport by plants (Zhu et al. 2025). In recent years, research focus has increasingly shifted from plant centered perspectives to rhizosphere processes, particularly the role of plant-microbe symbioses in enhancing stress tolerance and remediation efficiency (Liu et al. 2015).

Among rhizosphere microorganisms, arbuscular mycorrhizal fungi (AMF) represent a key functional group capable of forming mutualistic symbioses with the majority of terrestrial plants (Tan et al. 2015; Zhu et al. 2025). Extensive research indicates that AMF can regulate the speciation and bioavailability of heavy metals by modifying the rhizosphere microenvironment of host plants. For example, AMF can extend hyphae outward and alter soil pH and phosphatase activity, thereby influencing heavy metal dynamics (He et al. 2020). AMF can also influence the dissolution-precipitation equilibrium of heavy metals by altering rhizosphere pH and may reduce metal uptake into plant tissues through adsorption onto fungal hyphae (Zhuo et al. 2020; Riaz et al. 2021). Our research group previously demonstrated that AMF inoculation significantly increased biomass and Cd uptake in *S. calendulacea* (Lu et al. 2020). However, the effects of AMF on heavy metal bioavailability in the rhizosphere are highly context-dependent (Coninx et al. 2017). In particular, systematic comparative studies examining how AMF differentially regulate Cd dynamics in the rhizosphere and plant nutritional status across species with contrasting ecological backgrounds remain limited. This gap constrains the effective application of AMF–plant symbioses in targeted phytoremediation strategies.

Research has found that in Cd-contaminated soils, Cd disrupts the inherent ability of plants to acquire phosphorus (P) by inhibiting root growth and metabolic activity (Rehman et al. 2017), often resulting in reduced P availability. Therefore, there is an urgent need to explore external strategies to enhance plants P acquisition capacity (Duan et al. 2023). In this context, the symbiotic association between plants and AMF represents a promising biological approach. Beyond directly acquiring P by expanding root absorption areas, AMF enhance P availability by increasing soil phosphatase activity in the rhizosphere and promoting the mineralization of organic P (Akhtar et al. 2024; Khan et al. 2025). This strategy not only enables AMF to help plants cope with P starvation under Cd stress but may also, in turn, alter Cd dynamics within the soil-plant system. Changes in phosphatase activity are closely associated with rhizosphere pH and organic acid secretion, collectively forming a complex network that influences heavy metal speciation (Wang et al. 2025). Moreover, AMF regulation of plant mineral nutrition extends far beyond P, encompassing the absorption and redistribution of macroelement such as sodium (Na), potassium (K), calcium (Ca), and magnesium (Mg), as well as microelement including copper (Cu), zinc (Zn), iron (Fe), and manganese (Mn) (Zhao et al. 2024; Khoza et al. 2025). Dynamic changes in these elements are not only essential for plant physiological processes and stress tolerance but also play a critical role in Cd uptake, transport, and detoxification through ion antagonism, competition, and synergistic interactions. For example, Zn, Fe, and Cd uptake often share transporters, while Ca signaling pathways participate in Cd stress responses (Bozzi and Gaudet 2021). Therefore, systematically elucidating the coordinated changes in rhizosphere pH, Cd availability, phosphatase activity, and multi-element uptake mediated by AMF is essential for understanding the multifaceted mechanisms underlying AMF-enhanced Cd tolerance in plants.

Our team has previously demonstrated that hybrids derived from crosses between *Sphagneticola trilobata* and *Sphagneticola calendulacea* can tolerate high Cd environments through coordinated adjustments in photosynthetic performance, antioxidant capacity, leaf hormone regulation, and active detoxification strategies (Gao et al. 2022; Zhang et al. 2020). However, no studies have yet examined changes in Cd tolerance following inoculation with AMF. Although previous studies have individually examined the effects of AMF on plant growth, heavy metal uptake, or individual nutrient elements, comprehensive comparative analyses integrating rhizosphere chemical properties, key enzyme activities, and whole-plant mineral nutrient uptake (in both shoots and roots) across different plant ecotypes remain lacking. Such an integrated approach is essential for elucidating the mechanistic links among rhizosphere processes, nutrient acquisition, and plant responses to heavy metal stress under AMF regulation. Accordingly, the present study used native species (*S. calendulacea*), invasive species (*S. trilobata*), and their hybrid as materials. Under a Cd gradient, we hypothesize that inoculation with *Funneliformis mosseae* (FM) may mitigate cadmium toxicity in *Sphagneticola* by: (1) Modifying rhizosphere pH and enhancing phosphatase activity, thereby improving P acquisition; (2) Altering ion absorption and transport to prioritize the uptake and accumulation of certain essential elements while adjusting the competitive relationship between Cd and other metal cations, thereby maintaining ionic homeostasis and mitigating Cd toxicity. This study aims to elucidate the mechanism by which FM alleviates Cd stress through regulation of the rhizosphere microenvironment and coordinated nutrient management. It further evaluates the Cd remediation potential of the *Sphagneticola* under FM assistance, providing crucial theoretical foundations and germplasm resource selection references for targeted soil remediation utilizing plant FM symbiotic systems.

## Materials and methods

### Study subjects

The soil used in this experiment was collected from the Biological Garden of South China Normal University. Its basic physicochemical properties were as follows: pH 6.49 (1:2.5 w/v water), organic matter content of 1.75%, total nitrogen content of 1.32 g kg⁻¹, available potassium 83.15 mg kg⁻¹, available P 22.35 mg kg⁻¹, and Cd 0.05 mg kg⁻¹. After air-drying, the soil was ground, passed through a 2 mm sieve to remove stones and plant roots, and then sterilized at 121 °C (0.1 MPa) for 2 h. Subsequently, CdCl₂ was added to the soil at concentrations of 0, 25, 50, and 100 mg Cd kg⁻¹ respectively. To ensure uniform distribution, the Cd amended soils were saturated with deionized water for one week and air-dried in a cool, ventilated room for two weeks, this wetting-drying cycle was repeated three times. The final measured total Cd concentrations in the soil were 0.04, 25.02, 50.03, and 100.01 mg kg⁻¹, respectively.

The FM used in this experiment was obtained from the International Culture Collection of (Vesicular) Arbuscular Mycorrhizal Fungi (INVAM) and have been reported to exhibit strong resistance to Cd (Li et al. 2016). The AMF inoculum was propagated using corn as the host plant, cultivated in 3-liter pots filled with a sterile soil and sand (1:1, v/v). After five months, the colonized roots were harvested, cut into fragments, and uniformly mixed with a medium containing mycelia and spores. This mixture served as the AMF inoculum.

The three *Sphagneticola* species (including: *S. calendulacea*, *S. trilobata* and their hybrid) of chrysanthemum used in this experiment were obtained from the School of Life Sciences at South China Normal University (collected from the South China Botanical Garden of the Chinese Academy of Sciences and cultivated at the School of Life Sciences, South China Normal University). Healthy and mature stems from the three *Sphagneticola* species were cut into 8–10 cm stem segments, each retaining two nodes. The stem segments were then placed in dark culture bottles to promote rooting and shoot emergence.

### Experimental design

The experiment was set up with four treatment groups: 0 (Cd0), 25 (Cd25), 50 (Cd50), and 100 (Cd100) mg Cd kg⁻¹. For the potted plant trials involving FM inoculation, the soil was inoculated with the mycorrhizal inoculum at a concentration of 5% (w/w). Sterile inoculum (autoclaved at 121 °C for 2 h) was prepared as an uninoculated control. To compensate for the loss of soil bacteria caused by sterilization, 60 mL of filtrate (obtained by passing a suspension of non-sterilized soil through an 11 μm mesh) was added to each uninoculated control pot (Zhong et al. 2012). Uniform and vigorous seedlings of *S. calendulacea*, *S. trilobata*, and their hybrid (15–20 cm in height, with 5–6 pairs of leaves) were selected and planted in pots containing the four soil mixtures (top diameter 17.4 cm, bottom diameter 12.8 cm, height 14.7 cm), with 3 kg of soil per pot. One seedling was planted per pot, with five biological replicates (*n* = 5). The pots were randomly arranged in the greenhouse, rotating their positions weekly. Maintain all plants in a greenhouse at 21–29 °C with 14 h of daily light. The soil was moistened with sterile water to maintain 62% of the maximum water-holding capacity. After three months of cultivation, harvest all plants and separated into leaves, stems, and roots. Immerse the roots in 0.02 M EDTA for 45 min, then rinse with deionized water to remove metals adsorbed on the root surface. Plant samples for biomass and Cd concentration determination were dried in an oven to constant weight. The remaining fresh plant samples were immediately frozen in liquid nitrogen and stored at − 80 °C for later use (Jiang et al. 2019).

### Mycorrhizal colonization

Following the method described by Phillips and Hayman (1970), the mycorrhizal colonization rate was determined using staining and microscopic examination. Fresh fine root samples were randomly selected from each pot, carefully rinsed with distilled water to remove any adhering soil, and then cut into 1 cm long segments. The root segments were treated with a 10% KOH solution in a 90 °C water bath for 1 h until they became transparent, then rinsed thoroughly with deionized water and soaked in a 1% hydrochloric acid solution for 3 min. After washing, the root segments were stained with 0.05% trypan blue, and finally destained with a lactate–glycerol solution (v/v = 1:1). The stained root segments were examined under a microscope. The mycorrhizal colonization rate was calculated for 50 root segments from each pot using the grid counting method described by Giovannetti and Mosse (1980).

### Determination of P element and other macro- and microelement contents

After drying the harvested samples to constant weight, the samples were ground and passed through a sieve. Approximately 0.1 g of each sample was accurately weighed, and 5 mL of concentrated HNO₃ was added for digestion in a microwave digestion system (COOLPEX, Yiyao Technology, Shanghai). After digestion, the solution in the digestion vessel was filtered through a 0.22 μm membrane filter and diluted to a final volume of 10 mL. The Cd concentration was determined using an inductively coupled plasma mass spectrometer (ICP-MS) (Agilent 7800, USA). The standard curve [GBW07602 (GSV-1)] was purchased from the Beijing Research Center for Standard Materials, China.

### Determination of soil phosphatase activity

Approximately 1.00 g of fresh soil (< 2 mm) was weighed into a 50 mL centrifuge tube. Add 0.25 mL of toluene, 4.00 mL of MUB buffer (pH 6.5) for acid phosphatase, and 1 mL 4 nitrophenyl phosphate disodium salt solution prepared in the same buffer were added. Mix thoroughly and incubate at 37 °C for 1 h. After incubation, 4 mL of calcium chloride solution and 4 mL of sodium hydroxide solution were added. Mix thoroughly, filter, and measure the absorbance at 400 nm.

### Soil pH determination

After air drying and screening of rhizosphere soil, it was extracted with 0.01 M CaCl_2_ at 1:2.5 (soil: solution), equilibrated for 30 min, and the pH of the supernatant was measured using a pH meter.

### Determination of available cadmium in rhizosphere soil

The available Cd content in rhizosphere soil was determined using the DTPA (diethylenetriamine pentaacetic acid) extraction method with slight modifications (Feng et al. 2005). Soil samples from the rhizosphere of three *Sphagneticola* species were collected, air-dried, and sieved. Rhizosphere soil and DTPA extractant (0.005 M DTPA, 0.1 M triethanolamine, and 0.01 M CaCl₂ pH 7.3 ± 0.2) were added to conical flasks at a 1:2 (w: v) ratio. The mixture was stirred at room temperature for 2 h, then centrifuged, and the supernatant volume was adjusted to a fixed volume. The Cd concentration in the solution was determined by inductively coupled plasma mass spectrometry (ICP-MS).

### Statistical analysis

Experimental data were analyzed using IBM SPSS Statistics 19.0 (IBM, Armonk, NY, USA). All data are expressed as mean ± standard error (Mean ± SE). Duncan’s multiple range test was employed to assess significant differences between the control and treatment groups, with a significance level set at 0.05. All bar charts and line graphs were generated using SigmaPlot 14.0 software.

## Result

### Effects of FM on pH and DTPA in the rhizosphere soil of three *Sphagneticola* species

**Fig. 1:**
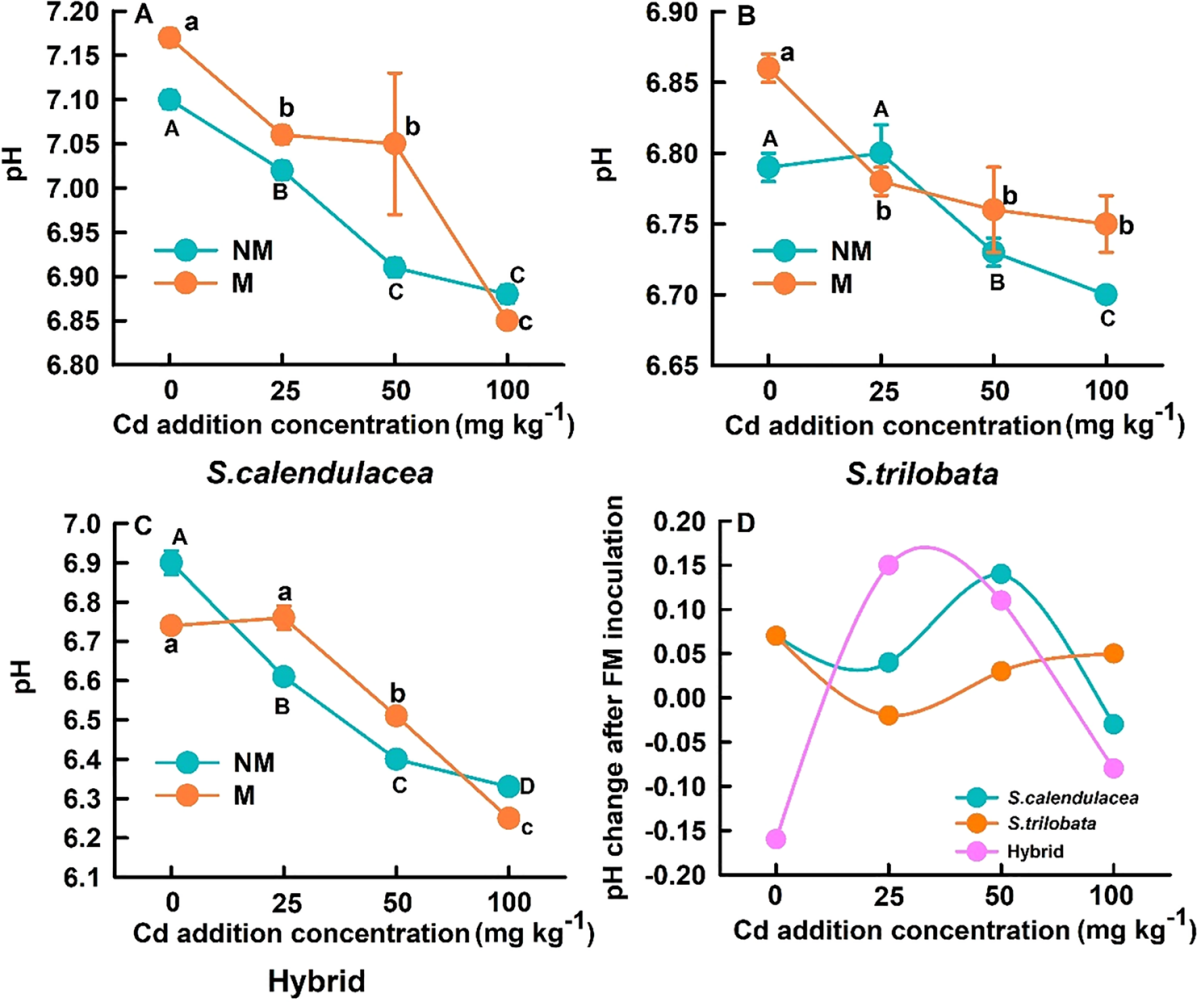
Effects of FM inoculation on pH in the rhizosphere soil of *S. calendulacea*, *S. trilobata*, and Hybrid (**A**-**C**); Changes in pH of rhizosphere soil around three *Sphagneticola* species following FM inoculation, as a function of Cd addition concentration (**D**). Different uppercase letters indicate significant differences in pH following the no FM inoculation treatment at different Cd concentrations. Different lowercase letters indicate significant differences in pH following the FM inoculation treatment at different Cd concentrations. All data are presented as mean ± standard error (SE) (*n* = 5)

The pH of the rhizosphere soil for native species (*S. calendulacea*), invasive species (*S. trilobata*), and their hybrid decreased significantly with increasing soil Cd concentration. Among them, the decrease was most pronounced in the hybrid, followed by *S. calendulacea*, with the smallest decrease observed in *S. trilobata* (Fig. 1). Similarly, after inoculation with FM, the pH of the rhizosphere soil for all three *Sphagneticola* species decreased with increasing soil Cd concentration. The magnitude of this decline for each *Sphagneticola* species followed the same trend as that observed in the non-inoculated treatments (Fig. 1A-C). Interestingly, at Cd concentrations of 0 mg Cd kg⁻¹, inoculation with FM resulted in higher pH values in the rhizosphere soil of *S. calendulacea* and *S. trilobata* compared to the non-inoculated controls, while the opposite was true for the hybrid (Fig. 1A-C). Compared to non-inoculated controls, FM inoculation induced distinct pH changes in the rhizosphere soils of the three *Sphagneticola* species. With the exception of the Cd25 and Cd50 mg kg^− 1^ treatments, where there were no significant differences in the *S. trilobata*, significant differences were detected in all other cases (Fig. 1A-C). Specifically, *S. calendulacea* showed an initial decrease followed by an increase and then a subsequent decrease; *S. trilobata* exhibited a decrease followed by an increase; and the hybrid demonstrated an increase followed by a decrease (Fig. 1D). In addition, following inoculation with FM under Cd100 conditions, the pH of the rhizosphere soil increased in *S. trilobata*, whereas the opposite was observed in *S. calendulacea* and the hybrid.

**Fig. 2:**
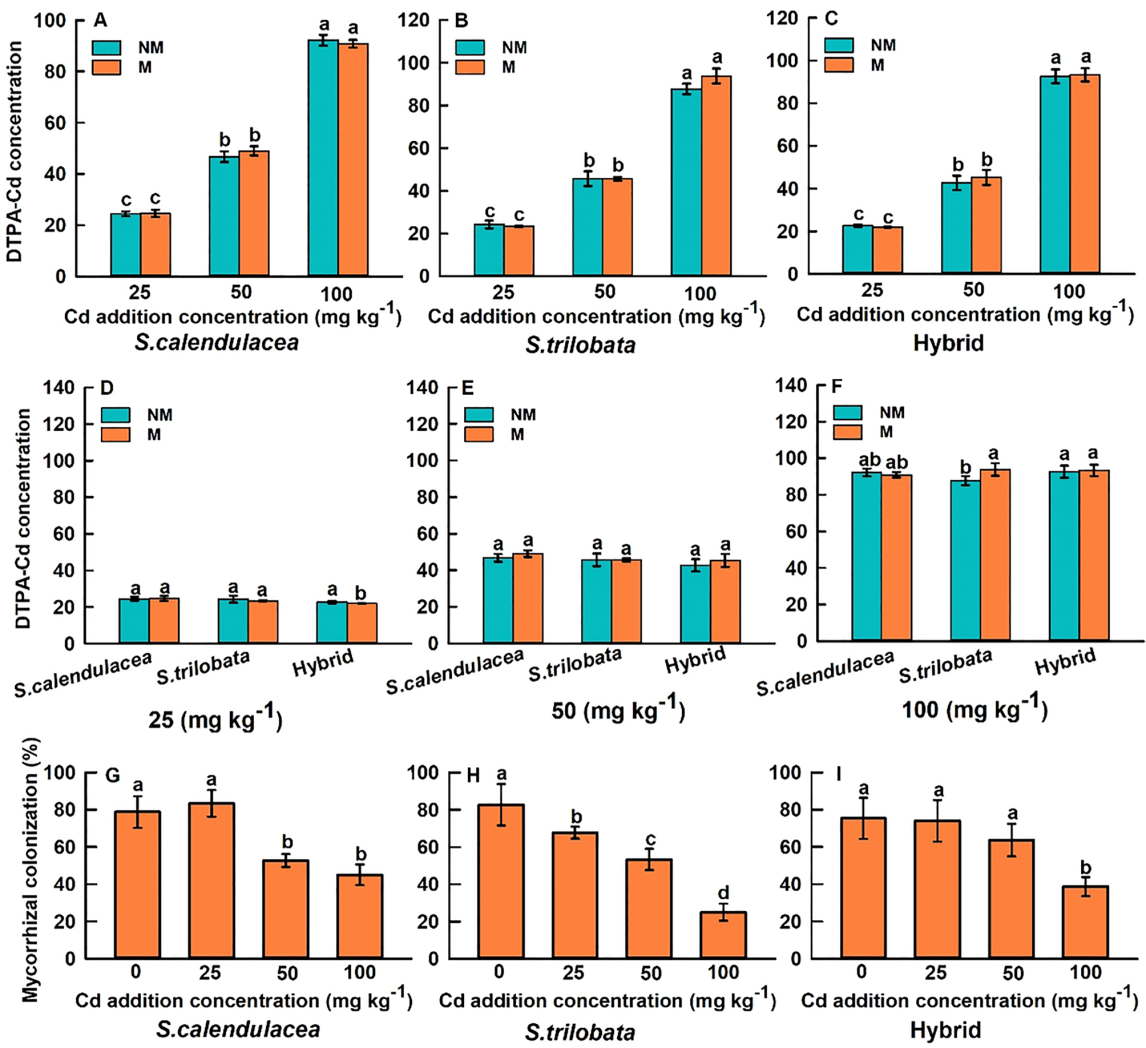
The changes in DTPA content in *S. calendulacea*, *S. trilobata*, and Hybrid grown in rhizosphere soils supplemented with various Cd concentrations (**A**-**C**). Changes in DTPA content in rhizosphere soil at identical Cd concentrations for *S. calendulacea*, *S. trilobata*, and Hybrid (**D**-**F**). The changes in colonization rate in *S. calendulacea*, *S. trilobata*, and Hybrid with various Cd concentrations (**G**-**I**). All data are presented as mean ± standard error (SE) (*n* = 5). Different letters above bars indicate statistical significance (*P* < 0.05)

As soil Cd concentration increased, the extractable Cd concentration in the rhizosphere soil of the three *Sphagneticola* species also increased significantly. For the same species, under identical Cd concentration gradients, inoculation with FM did not significantly alter extractable Cd concentration in the rhizosphere soil compared to the non-inoculated controls (Fig. 2). However, when comparing all three plants collectively, it was observed that at a Cd concentration of 25 mg kg^− 1^, the extractable Cd concentration in the rhizosphere soil of the hybrid inoculated with FM was significantly lower than that of the non-inoculated control (Fig. 2D). Conversely, at a Cd concentration of 100 mg kg^− 1^, the extractable Cd concentration in the rhizosphere soil of the *S. trilobata* was significantly higher than that of the non-inoculated control (Fig. 2F). As soil Cd concentrations increased, the mycorrhizal colonization rates of the three *Sphagneticola* species showed a significant decreasing trend; however, there were differences in how the various species responded to Cd stress. Specifically, the colonization rate of the *S. calendulacea* was highest at Cd concentrations of 0 and 25 mg kg^− 1^, but decreased significantly at 50 and 100 mg kg^− 1^(Fig. 2G). The colonization rate of *S. trilobata* showed a continuous decline as Cd concentration increased; in contrast (Fig. 2H); The colonization rate of hybrid did not differ significantly at Cd concentrations of 0, 25, and 50 mg kg^− 1^, and showed a significant decrease only at the 100 mg kg^− 1^ treatment (Fig. 2I).

### Effects of FM on P content in three *Sphagneticola* species organs

**Fig. 3:**
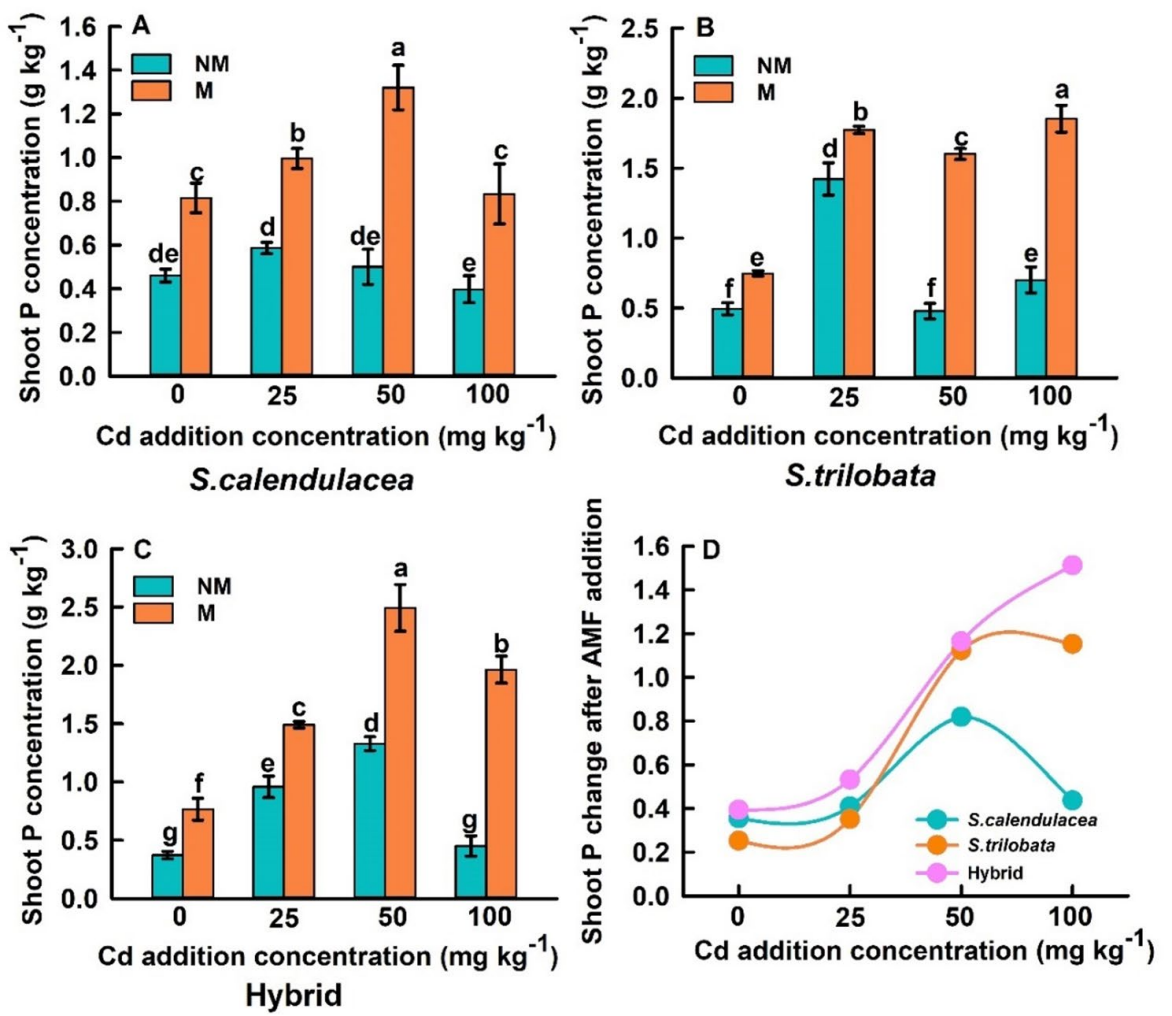
Effects of FM inoculation on P content in the shoot parts of *S. calendulacea*, *S. trilobata*, and Hybrid (**A**-**C**); Changes in P content in the shoot parts of three *Sphagneticola* species following FM inoculation, as a function of Cd addition concentration (**D**). Data source of *S. calendulacea* (**A**) Lu et al. (2020). All data are presented as mean ± standard error (SE) (*n* = 5). Different letters above bars indicate statistical significance (*P* < 0.05)

This study investigates the changes in P content in the shoots and roots of three *Sphagneticola* species under different Cd concentration gradients after inoculation with FM. FM inoculation significantly enhanced P uptake in both shoots and roots of *S. calendulacea*, *S. trilobata*, and the hybrid. The P content in both shoots and roots of the three *Sphagneticola* species exhibited a trend of initial increase followed by a decrease with increasing Cd concentrations. Under Cd0, Cd25, Cd50, and Cd100 conditions, compared to non-inoculated controls, the P content in the shoots of *S. calendulacea* increased by 77.14%, 69.76%, 163.56%, and 109.81%, respectively, while the P content in the roots increased by 183.89%, 74.92%, 82.99%, and 110.64%, respectively. For *S. trilobata*, shoot P content increased by 51.06%, 24.70%, 234.82%, and 164.40%, respectively, while root P content increased by 158.82%, 26.74%, 34.97%, and 156.80%, respectively. The P content in the shoots of the hybrid increased by 105.89%, 55.45%, 87.68%, and 335.31%, respectively, while the P content in the roots increased by 235.01%, 55.49%, 46.17%, and 133.33%, respectively (Fig. 3).

### Effects of FM on phosphatase activity in rhizosphere soil of three *Sphagneticola* species

FM inoculation enhanced acid phosphatase activity in the rhizosphere soil of *S. calendulacea*, *S. trilobata*, and the hybrid (Fig. 5A-C). Furthermore, both *S. calendulacea* and the hybrid exhibited significantly increased acid phosphatase activity in the rhizosphere soil with increasing soil Cd concentration (Fig. 5A, C). Under Cd0 and Cd50 conditions, rhizosphere soil acid phosphatase activity in *S. calendulacea* increased by 14.32% and 23.80%, respectively, compared to non-inoculated controls. In *S. trilobata*, acid phosphatase activity in the rhizosphere soil increased by 19.75% and 15.23%, respectively, compared to the non-inoculated controls. Similarly, in the hybrid, acid phosphatase activity in the rhizosphere soil increased by 6.03% and 21.33%, respectively, compared to the non-inoculated controls. Following inoculation with FM, the alkaline phosphatase activity in the rhizosphere soil of the three *Sphagneticola* species did not exhibit a clear pattern. Specifically, under Cd0 conditions, no significant differences in alkaline phosphatase activity were observed among the three *Sphagneticola* species. Under Cd50 conditions, AMF inoculation significantly decreased alkaline phosphatase activity in *S. calendulacea*, significantly increased it in *S. trilobata*, and did not significantly affect it in the hybrid (Fig. 5D-F).

### Effects of FM on the absorption of macroelements and microelements in three *Sphagneticola* species

Table 1 shows the changes in the macronutrient content of Na, Mg, K, and Ca in the shoots and roots of three *Sphagneticola* species after inoculation with FM under different Cd concentration gradients. Under Cd0, Cd25, Cd50, and Cd100 conditions, compared to the non-inoculated controls, FM inoculation primarily promoted the uptake of Na in the roots of *S. calendulacea* and the hybrid. A similar trend was observed in *S. trilobata*, except under Cd100 treatment. In contrast, under Cd0, Cd25, Cd50, and Cd100 conditions, compared with the Na content in the non-inoculated controls, inoculation with FM reduced Na uptake in the shoot parts of *S. calendulacea* (except under Cd50 conditions) and *S. trilobata*. In addition, after inoculation with FM, Mg content in both the shoots and roots of *S. calendulacea*, *S. trilobata*, and the hybrid generally increased under Cd0, Cd25, Cd50, and Cd100 conditions; however, no significant differences were observed at certain Cd concentrations. The shoots include: *S. calendulacea* under Cd100 conditions and *S. trilobata* under Cd50 conditions; the roots include: *S. calendulacea* under Cd0, Cd25, Cd50, and Cd100 conditions; *S. trilobata* under Cd100 conditions; and the hybrid under Cd50 conditions. However, inoculation with FM promoted K uptake in the shoots of *S. calendulacea*, *S. trilobata*, and the hybrid, while simultaneously reducing K uptake in the roots of all three *Sphagneticola* species under Cd0, Cd25, Cd50, and Cd100 conditions, with the exception of *S. calendulacea* grown under Cd100 conditions and the hybrid grown under Cd100 conditions. Additionally, inoculation with FM promoted Ca uptake in the shoot parts of *S. calendulacea*, *S. trilobata*, and the hybrid in most treatments, while Ca concentrations decreased in some treatment groups.

**Table 1:** The Na、Mg、K、Ca concentrations in three *Sphagneticola* plants grown in soils supplemented with various Cd concentrations

	(mg kg^− 1^)		Na (g kg^− 1^, DW)		Mg (mg kg^− 1^, DW)		K (g kg^− 1^, DW)		Ca (g kg^− 1^, DW)	
			Shoot	Root	Shoot	Root	Shoot	Root	Shoot	Root
*S.calendulacea*	0	NM	4.69 ± 0.17a	5.39 ± 0.31a	347.42 ± 17.10a	83.59 ± 7.65b	14.19 ± 0.74 cd	14.55 ± 1.93 cd	6.47 ± 0.66a	3.16 ± 0.24a
		M	6.80 ± 0.77b	11.95 ± 0.57f	395.99 ± 4.55a	105.05 ± 3.36c	18.43 ± 1.49e	8.43 ± 1.19a	6.19 ± 0.50a	4.36 ± 0.29 cd
	25	NM	11.63 ± 0.42f	6.59 ± 0.52b	632.76 ± 49.38b	73.14 ± 6.61ab	12.40 ± 0.91bc	15.28 ± 1.94d	10.66 ± 0.46 cd	4.14 ± 0.27bcd
		M	9.07 ± 0.83de	10.78 ± 0.89e	891.18 ± 96.25 cd	84.98 ± 6.86b	17.15 ± 0.64e	8.31 ± 0.57a	8.70 ± 0.68b	3.82 ± 0.25bc
	50	NM	8.43 ± 0.76cde	6.17 ± 0.29ab	775.99 ± 55.19c	72.89 ± 7.16ab	11.86 ± 1.47b	14.92 ± 1.23 cd	9.79 ± 0.59c	3.79 ± 0.15b
		M	7.87 ± 0.32bcd	11.95 ± 0.82f	1192.52 ± 150.55f	82.82 ± 6.25b	16.86 ± 1.58e	9.26 ± 1.15a	10.29 ± 0.97c	4.21 ± 0.23bcd
	100	NM	9.27 ± 1.14e	8.43 ± 0.68c	940.63 ± 78.28de	68.00 ± 6.95a	9.93 ± 0.07a	12.68 ± 0.65bc	11.42 ± 0.42d	4.16 ± 0.19bcd
		M	7.56 ± 0.73ab	9.46 ± 0.24d	1034.96 ± 67.77e	78.40 ± 6.17ab	14.35 ± 0.50d	11.91 ± 0.54b	10.83 ± 0.32 cd	4.48 ± 0.54d
*S. trilobata*	0	NM	4.14 ± 0.17ab	4.78 ± 0.70a	880.95 ± 145.23b	96.40 ± 3.14b	12.11 ± 0.55a	15.39 ± 1.09d	9.27 ± 0.74b	3.42 ± 0.16a
		M	4.12 ± 0.21ab	14.74 ± 0.60e	1272.84 ± 79.00d	114.73 ± 0.25c	13.27 ± 0.44a	6.74 ± 0.66a	6.40 ± 0.12a	4.20 ± 0.18a
	25	NM	3.83 ± 0.43a	12.62 ± 1.31d	1241.52 ± 159.65d	100.10 ± 1.93b	22.48 ± 1.48c	13.94 ± 1.28 cd	9.58 ± 0.62b	4.46 ± 0.32a
		M	5.02 ± 0.21c	16.92 ± 0.62f	1556.08 ± 75.12e	114.47 ± 3.93c	29.26 ± 1.18e	6.10 ± 0.84a	11.54 ± 0.36d	3.82 ± 0.17a
	50	NM	7.00 ± 0.82d	5.37 ± 0.23a	1008.25 ± 37.19bc	78.70 ± 4.41a	16.45 ± 0.34b	15.19 ± 0.52d	10.42 ± 0.75c	4.15 ± 0.41a
		M	5.16 ± 0.61c	9.99 ± 0.65c	1160.03 ± 134.86 cd	99.01 ± 8.56b	26.41 ± 1.18d	9.11 ± 0.61b	11.09 ± 0.18 cd	4.66 ± 1.14ab
	100	NM	8.26 ± 0.89e	8.44 ± 0.90b	397.94 ± 38.57a	85.46 ± 8.82ab	15.71 ± 1.83b	15.41 ± 1.89d	9.24 ± 1.07b	5.06 ± 0.53ab
		M	4.80 ± 0.35bc	9.64 ± 1.32bc	1223.78 ± 187.41 cd	96.37 ± 18.87b	25.27 ± 1.68d	13.10 ± 1.32c	11.68 ± 0.44d	6.34 ± 1.34b
Hybrid	0	NM	3.51 ± 0.20a	4.86 ± 0.42a	397.09 ± 51.05a	67.31 ± 5.05ab	11.29 ± 0.62a	10.55 ± 0.69c	5.98 ± 0.26	3.00 ± 0.17a
		M	7.35 ± 0.18c	16.37 ± 1.19d	904.33 ± 44.15bc	112.70 ± 7.06c	14.34 ± 0.99b	5.40 ± 0.51a	5.53 ± 0.34	4.27 ± 0.40c
	25	NM	6.89 ± 0.21c	9.13 ± 0.43b	710.64 ± 69.96b	79.22 ± 10.64b	17.28 ± 1.64c	11.88 ± 1.31 cd	8.27 ± 0.29	4.39 ± 0.67c
		M	11.21 ± 0.42f	18.50 ± 0.89e	1517.29 ± 63.33d	101.20 ± 10.56c	21.22 ± 1.64d	8.53 ± 0.98b	8.24 ± 0.41	3.75 ± 0.37bc
	50	NM	6.26 ± 0.30b	9.43 ± 0.79b	1087.14 ± 44.73c	75.62 ± 11.03b	23.96 ± 1.91e	16.56 ± 1.18f	9.08 ± 0.14	4.16 ± 0.31c
		M	9.03 ± 0.19e	13.79 ± 1.50c	2512.50 ± 80.71e	81.54 ± 16.72b	30.25 ± 2.16f	11.91 ± 1.58 cd	13.04 ± 0.35	4.01 ± 0.22bc
	100	NM	5.71 ± 0.33b	5.69 ± 0.43a	1510.79 ± 163.69d	53.95 ± 7.17a	14.18 ± 1.20b	14.53 ± 1.00e	13.82 ± 1.13	3.44 ± 0.31ab
		M	7.99 ± 0.56d	13.42 ± 0.74c	2792.87 ± 311.73f	77.08 ± 0.69b	23.55 ± 0.90de	13.57 ± 0.94de	17.34 ± 1.01	4.11 ± 0.26bc

Note: Values are the means ± SDs (*n* = 5); Values with different letters within the same column denote significant differences at the *p* < 0.05 level

Table 2 shows the changes in micorelement Cu, Fe, Zn, and Mn content in the shoots and roots of three *Sphagneticola* species after inoculation with FM under different Cd concentration gradients. Under Cd0, Cd25, Cd50, and Cd100 conditions, FM inoculation promoted Cu uptake in the shoots and roots of *S. calendulacea*, *S. trilobata*, and the hybrid compared to the non-inoculated controls. A similar pattern was observed for Zn, except under Cd100 conditions. Inoculation with FM reduced Mn uptake in the shoot parts of *S. calendulacea*, *S. trilobata*, and the hybrid, while increasing Mn uptake in their roots. FM inoculation promoted Fe uptake in the roots of all three *Sphagneticola* species, whereas no consistent pattern was observed in the shoots.

**Table 2:** The Cu、Fe、Zn、Mn concentrations in three *Sphagneticola* plants grown in soils supplemented with various Cd concentrations

	(mg kg^− 1^)		Cu (mg kg^− 1^, DW)		Fe (mg kg^− 1^, DW)		Zn (mg kg^− 1^, DW)		Mn (mg kg^− 1^, DW)	
			Shoot	Root	Shoot	Root	Shoot	Root	Shoot	Root
*S.calendulacea*	0	NM	8.69 ± 0.44a	22.01 ± 0.99b	283.07 ± 17.27a	651.40 ± 76.83a	41.63 ± 3.56b	22.06 ± 1.59ab	69.25 ± 6.88e	17.84 ± 1.40c
		M	17.47 ± 1.26d	82.00 ± 6.49e	278.12 ± 10.39a	855.89 ± 72.23b	55.98 ± 4.65c	32.77 ± 2.73de	13.07 ± 1.81bc	8.84 ± 1.42ab
	25	NM	12.78 ± 1.14c	16.74 ± 1.56ab	291.58 ± 23.15a	830.48 ± 75.60ab	34.30 ± 2.46a	58.20 ± 5.74f	25.98 ± 2.91d	8.84 ± 0.24ab
		M	26.05 ± 1.18f	64.60 ± 7.50d	333.89 ± 21.70b	1510.90 ± 92.45d	42.29 ± 3.68b	38.79 ± 4.70e	9.40 ± 0.68ab	9.77 ± 0.61ab
	50	NM	10.71 ± 0.14b	17.08 ± 1.52ab	287.67 ± 32.01a	934.74 ± 128.71b	30.68 ± 0.86a	18.97 ± 1.82ab	14.33 ± 1.17bc	8.10 ± 0.81a
		M	22.18 ± 1.18e	70.50 ± 11.22d	288.28 ± 14.43a	1308.99 ± 60.10c	40.12 ± 3.31b	25.27 ± 4.52bc	6.00 ± 0.47a	8.63 ± 1.67ab
	100	NM	7.75 ± 0.44a	11.29 ± 1.34a	291.67 ± 14.26a	990.19 ± 94.57b	30.27 ± 1.54a	16.86 ± 1.41a	24.74 ± 0.16d	9.95 ± 1.56ab
		M	13.98 ± 0.58c	34.96 ± 1.72c	348.73 ± 24.48b	1755.85 ± 185.17e	33.12 ± 0.30a	30.15 ± 3.60 cd	15.86 ± 1.14c	10.57 ± 1.09b
*S. trilobata*	0	NM	16.55 ± 1.80b	56.66 ± 10.26b	267.18 ± 36.04a	1670.00 ± 90.44a	54.22 ± 4.03c	19.89 ± 1.15a	99.02 ± 4.28e	16.08 ± 1.67a
		M	21.80 ± 1.23c	192.34 ± 11.00d	286.94 ± 38.23ab	1817.47 ± 300.11a	63.62 ± 5.67d	28.77 ± 1.36c	18.91 ± 3.71c	15.17 ± 1.80a
	25	NM	19.66 ± 1.58bc	75.07 ± 6.91bc	257.28 ± 16.49a	3064.45 ± 140.56bc	47.96 ± 5.37bc	24.31 ± 1.23b	13.22 ± 1.32b	12.99 ± 1.69a
		M	26.21 ± 1.85d	186.58 ± 17.53d	277.75 ± 21.09ab	3870.99 ± 434.24d	61.24 ± 2.20d	28.93 ± 3.69c	8.73 ± 0.59a	13.04 ± 2.30a
	50	NM	10.26 ± 1.28a	21.30 ± 0.93a	340.34 ± 23.67c	2650.00 ± 150.00b	35.51 ± 1.29a	18.14 ± 0.85a	24.12 ± 1.01d	14.91 ± 5.46a
		M	31.10 ± 3.40e	85.75 ± 20.22c	313.02 ± 15.39bc	2718.80 ± 177.75b	52.20 ± 0.90c	26.76 ± 0.75bc	13.87 ± 1.29b	22.10 ± 2.81b
	100	NM	10.70 ± 0.85a	16.47 ± 1.40a	343.83 ± 30.03c	3367.14 ± 403.33c	41.94 ± 4.22b	42.28 ± 5.05d	23.80 ± 3.41d	16.62 ± 1.91a
		M	25.68 ± 1.62d	87.47 ± 13.91c	344.56 ± 12.93c	3898.21 ± 480.16d	60.51 ± 4.57d	50.46 ± 3.43e	14.86 ± 1.85b	26.76 ± 2.27c
Hybrid	0	NM	10.67 ± 1.22a	15.22 ± 1.07a	282.63 ± 27.30ab	876.30 ± 65.99a	65.36 ± 4.02c	22.70 ± 2.20ab	43.94 ± 4.29e	16.15 ± 2.06c
		M	17.73 ± 1.65c	207.18 ± 9.82 g	277.12 ± 3.77ab	1961.15 ± 116.17d	80.14 ± 4.48d	44.51 ± 4.01e	14.04 ± 0.97bc	15.43 ± 2.27bc
	25	NM	14.18 ± 1.36b	153.56 ± 14.69e	281.86 ± 19.84ab	1414.04 ± 123.25bc	49.58 ± 2.27b	28.86 ± 2.39c	11.81 ± 0.65ab	12.36 ± 1.47abc
		M	24.20 ± 1.08d	180.43 ± 9.78f	273.71 ± 13.70a	2271.88 ± 159.25e	65.66 ± 3.27c	35.52 ± 1.51d	9.00 ± 0.67a	13.55 ± 0.75abc
	50	NM	17.13 ± 0.96c	86.44 ± 11.76c	292.76 ± 25.83ab	1242.12 ± 101.28b	41.68±.46a	21.66 ± 1.24a	15.27 ± 1.08c	14.04 ± 2.23abc
		M	34.61 ± 1.96e	113.38 ± 7.69d	312.37 ± 16.38b	1518.48 ± 205.49c	64.76 ± 2.44c	24.08 ± 0.36ab	8.84 ± 0.38a	13.37 ± 1.77abc
	100	NM	10.46 ± 0.63a	14.88 ± 1.36a	290.43 ± 20.28ab	760.66 ± 96.61a	45.36 ± 5.06ab	24.63 ± 3.67abc	37.92 ± 1.32d	10.93 ± 1.81a
		M	24.09 ± 1.74d	63.20 ± 5.80b	299.22 ± 11.84ab	1940.96 ± 141.28d	62.70 ± 2.51c	27.04 ± 1.32bc	11.41 ± 0.68ab	11.77 ± 3.03ab

Note: Values are the means ± SDs (*n* = 5); Values with different letters within the same column denote significant differences at the *p* < 0.05 level

## Discussion

This study systematically investigated the effects of inoculation with the FM on the rhizosphere microenvironment, nutrient uptake, and tolerance mechanisms of three *Sphagneticola* species (*S. calendulacea*, *S. trilobata*, and their hybrid) under Cd stress. Results confirmed our initial hypothesis: FM significantly enhanced the tolerance of ragwort to Cd stress by synergistically regulating rhizosphere chemical processes and host plant nutrient homeostasis.

### Differential regulation of AMF on rhizosphere soil pH

Our research indicates that Cd stress generally reduces soil pH in the rhizosphere of three *Sphagneticola* species (Fig. 1), which is consistent with findings from numerous studies showing that heavy metal ions induce roots to secrete H⁺ to maintain ionic balance or activate tolerance mechanisms (Zhang et al. 2024). However, after inoculation with FM, changes in rhizosphere pH exhibited significant species dependence. In the absence of Cd stress, FM inoculation increased rhizosphere pH in the *S. calendulacea* and *S. trilobata*, but decreased it in the hybrid species (Fig. 1A-C). These differences may stem from inherent variations in root exudate patterns (such as organic acids) during the establishment of FM symbiosis across different plant species, as well as differences in the effects of mycelial networks on ion exchange processes (Kushwaha et al. 2015; Zhang et al. 2024). AMF hyphae themselves possess the ability to secrete protons and organic anions, and can influence the root secretion behavior of host plants, thereby jointly determining acidification or alkalization effects in the rhizosphere (Shen et al. 2011; Sessitsch et al. 2013; Wang et al. 2022). Under Cd stress gradients, plants inoculated with FM exhibited a similar pattern of pH decline to the non-inoculated group, but with different magnitudes of change. This indicates that FM did not reverse Cd induced acidification trends but likely participated in buffering and reshaping the rhizosphere environment by modulating the rate or extent of acidification processes (Fig. 1). Rhizosphere pH is a key factor controlling heavy metal dissolution, precipitation, adsorption, and complexation reactions (Rajkumar et al. 2012). Therefore, the species-specific changes in rhizosphere pH induced by FM constitute the primary microenvironmental basis for subsequent effects on Cd bioavailability and nutrient availability. It is worth noting that FM inoculation did not generally alter the DTPA-Cd content in the rhizosphere soil in this study (Fig. 2); however, significant differences were observed at specific high Cd concentrations (e.g., the hybrid under Cd25 conditions and the *S. trilobata* under Cd100 conditions). FM did not consistently increase or decrease the amount of Cd available to plants in the rhizosphere; this finding is partially consistent with conclusions from some studies suggesting that AMF reduce the availability of Cd to plants by adsorbing it via their hyphae, sequestering it within mycorrhizal structures, or indirectly influencing Cd speciation by altering rhizosphere pH (Rajkumar et al. 2012; He et al. 2020). Therefore, we speculate that the primary mechanism by which FM enhances the cadmium tolerance of *Sphagneticola* is not through altering Cd bioavailability in the soil, but rather through enhancing the plant own physiological tolerance mechanisms.

### FM significantly enhances plant P uptake and correlates with rhizosphere phosphatase activity

**Fig. 4:**
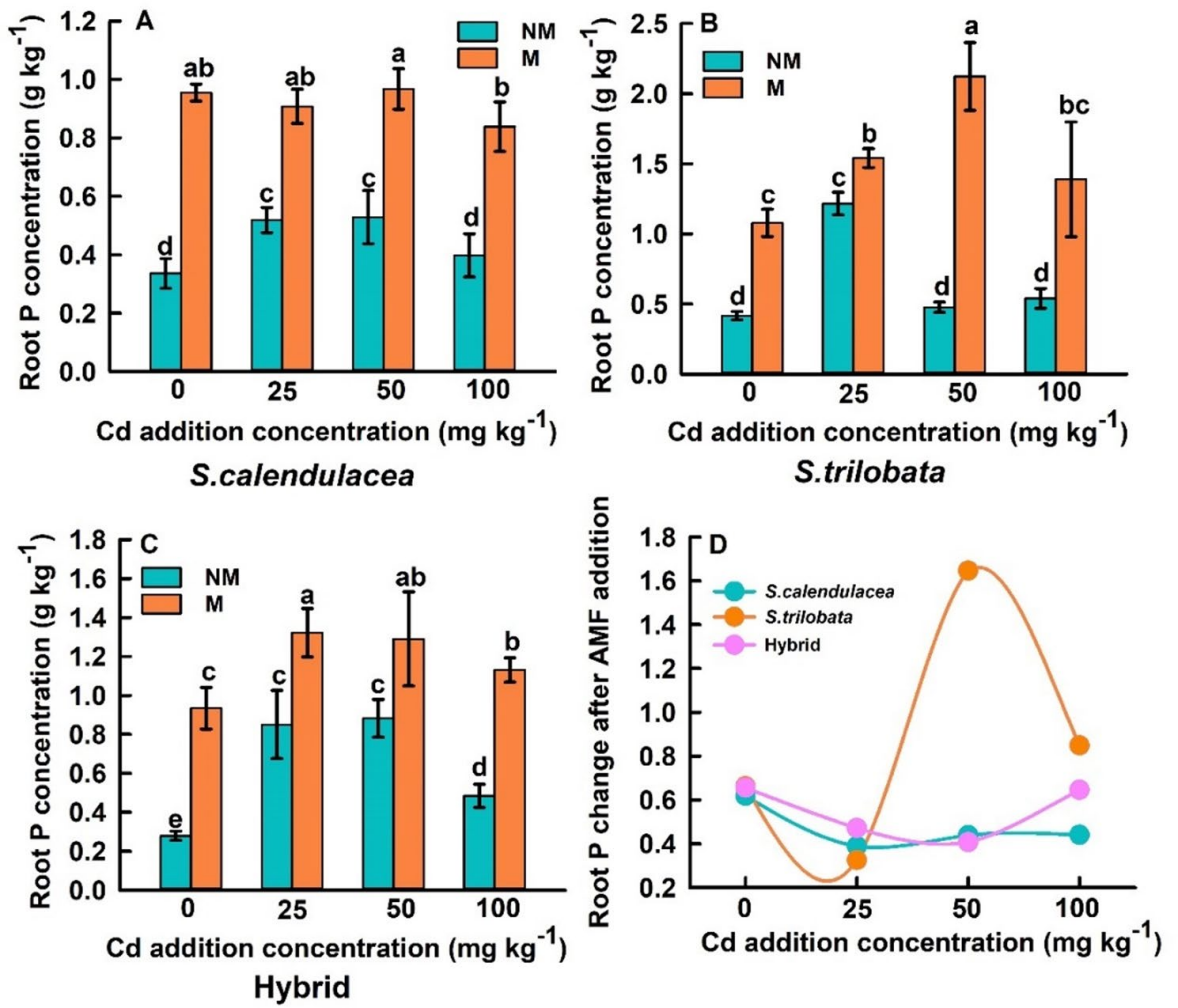
Effects of FM inoculation on P content in the root parts of *S. calendulacea*, *S. trilobata*, and Hybrid (**A**-**C**); Changes in P content in the root parts of three *Sphagneticola* species following FM inoculation, as a function of Cd addition concentration (**D**). Data source of *S. calendulacea* (**A**) Lu et al. (2020). All data are presented as mean ± standard error (SE) (*n* = 5). Different letters above bars indicate statistical significance (*P* < 0.05)

**Fig. 5:**
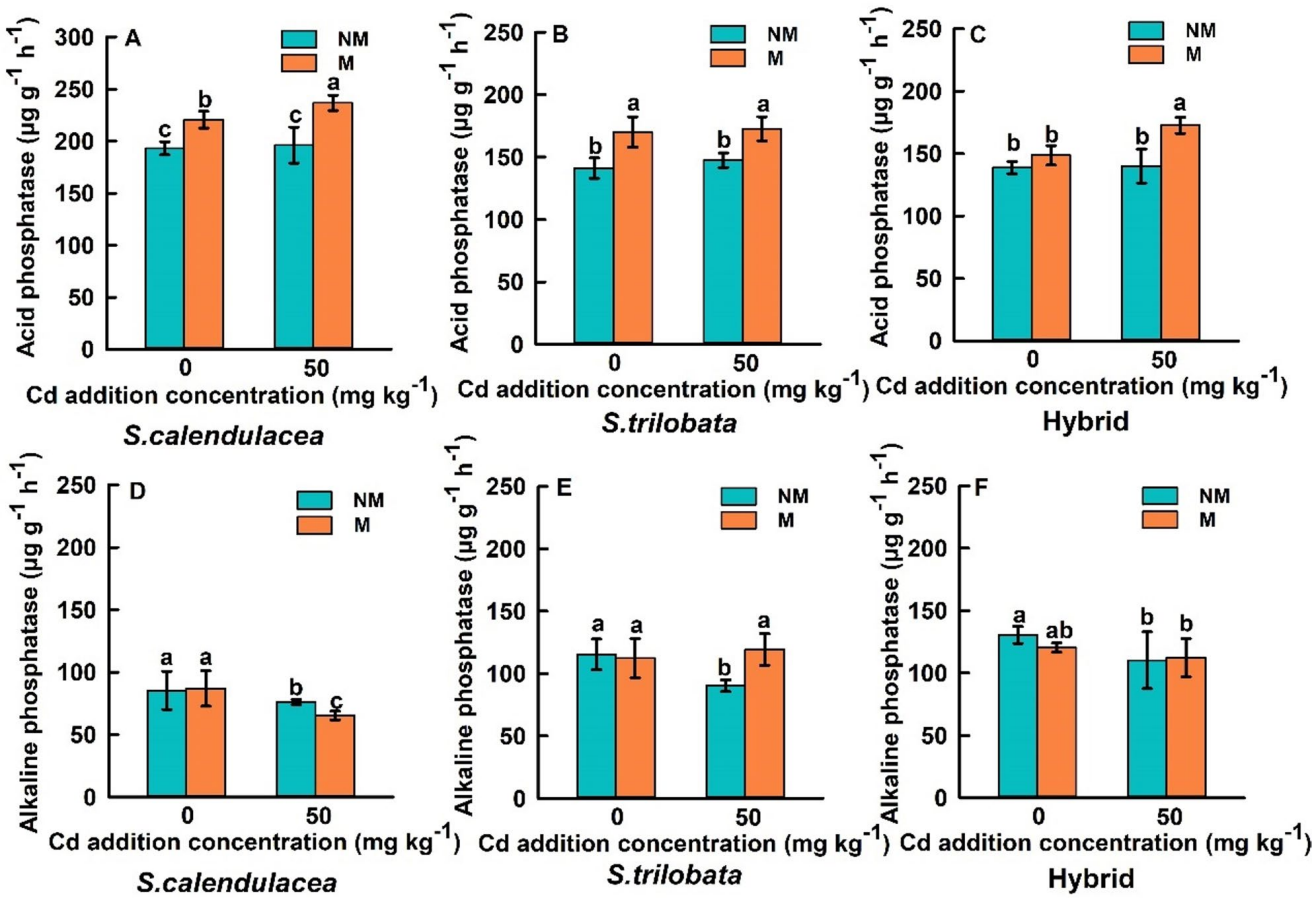
Effects of FM inoculation on acid phosphatase activity in the rhizosphere soil of *S. calendulacea*, *S. trilobata*, and Hybrid (**A**-**C**); Effects of FM inoculation on alkaline phosphatase activity in the rhizosphere soil of *S. calendulacea*, *S. trilobata*, and Hybrid (**D**-**F**). All data are presented as mean ± standard error (SE) (*n* = 5). Different letters above bars indicate statistical significance (*P* < 0.05)

One of the most significant findings of this study is that FM inoculation substantially enhanced P uptake in both the shoot and root parts of three *Sphagneticola* species, with increases generally exceeding 50%, which is consistent with the trends observed in our team previous research, which showed that FM inoculation significantly increased the uptake of phosphorus by *S. calendulacea* (Lu et al. 2020). This effect was particularly pronounced under high Cd stress (Figs. 3 and 4). This strongly supports our first hypothesis: FM assists plants in coping with Cd stress by enhancing their P acquisition efficiency. Cd stress typically disrupts root structure and function, inhibiting the plant’s own P uptake capacity and leading to physiological P starvation (Guo et al. 2013; Duan et al. 2023). AMF significantly expand the root absorption space through their extensive arbuscular mycelial networks (Wang et al. 2022). More importantly, we found that FM inoculation universally increased acid phosphatase (ACP) activity in the rhizosphere soil, with both *S. calendulacea* and the hybrid varieties exhibiting further enhanced ACP activity as Cd concentration increased (Fig. 5A-C). This reveals another key mechanism by which FM enhances plant P nutrition: by accelerating the mineralization of organic P in soil through the activation of phosphatases secreted by rhizosphere microorganisms, thereby increasing the pool of available P (An et al. 2022; Guo et al. 2023). Cd stress itself may act as an abiotic signal, stimulating the plant AMF symbiont system to intensify the mobilization of organic P resources, manifested as elevated ACP activity. However, the response of alkaline phosphatase (ALP) activity to FM inoculation was inconsistent and lacked clear patterns (Fig. 5D-F). This may be attributed to the acidic pH of the experimental soil, which favors ACP activity, and also reflects the complexity of different phosphatase sources and their regulatory mechanisms (Nannipieri et al. 2010; Karaca et al. 2009). The enhancement of ACP activity was strongly positively correlated with a significant increase in plant P content, confirming that AMF inoculation plays a central role in alleviating Cd induced P limitation by enhancing rhizosphere phosphatase activity (especially ACP), promoting organic P mineralization, and increasing plant P uptake.

### FM comprehensively regulates mineral element homeostasis to counteract Cd toxicity

**Fig. 6:**
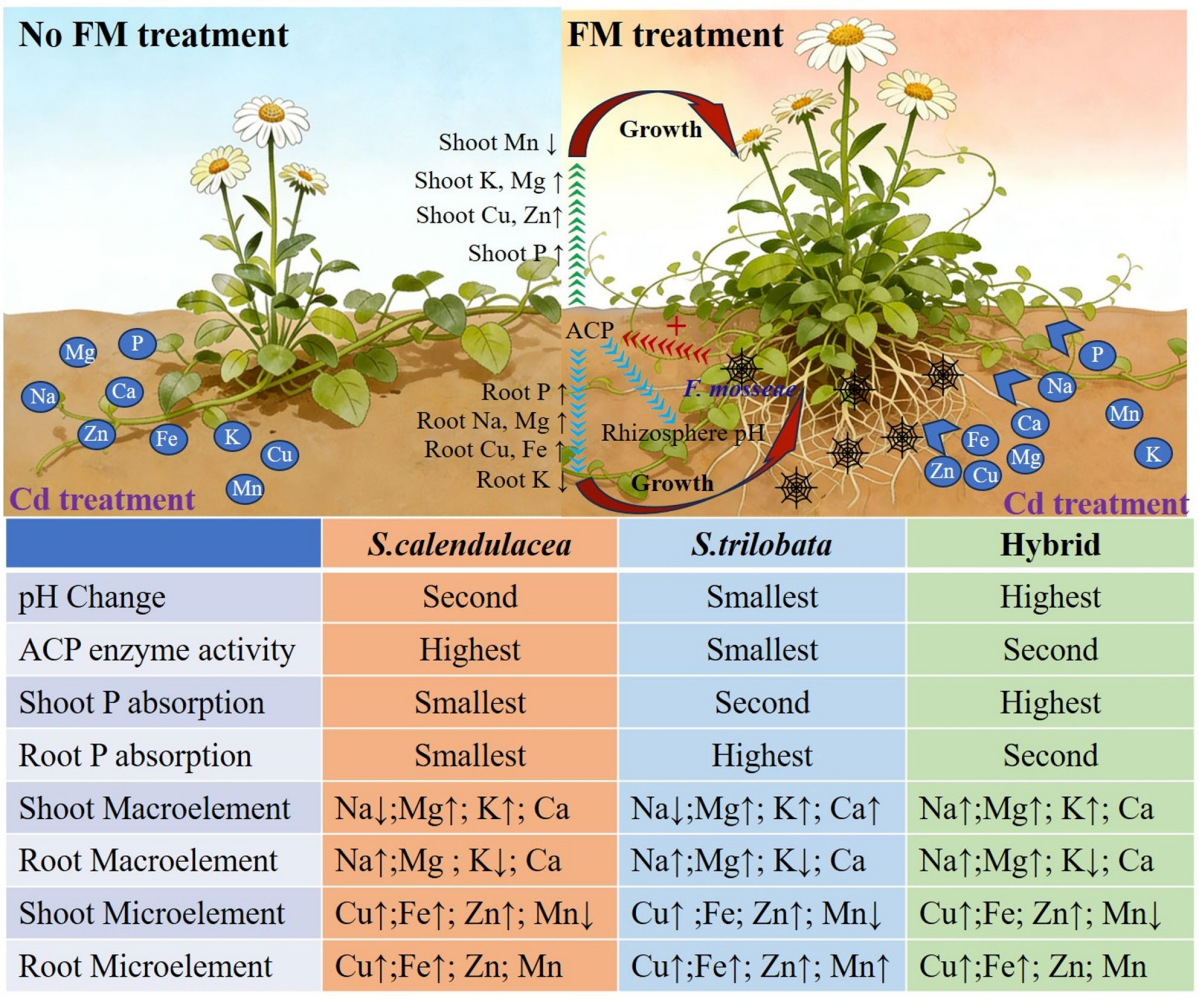
Conceptual diagram illustrating differences in rhizosphere microenvironment and macorelement uptake between three *Sphagneticola* species with and without FM inoculation. The up arrow indicates an increase. The down arrow indicates a decrease

Our second hypothesis that FM maintains homeostasis and mitigates Cd toxicity by adjusting ion uptake profiles is also supported by the data (Tables 1 and 2). Analysis of macronutrients and micronutrients indicates that FM inoculation exerts comprehensive and profound influences on the mineral nutrition of *Sphagneticola*. FM generally promoted the accumulation of Na, Mg, and Ca in roots while facilitating K transport to the shoots (increased K in shoots and decreased K in roots) (Fig. 6). Ca²⁺ as a crucial second messenger, participates in Cd stress perception and signal transduction in plants (Ren et al. 2022). Its elevated levels help maintain the stability of cell walls and membrane structures while competing for Cd binding sites (Liu et al. 2023). K⁺ also competes with Cd²⁺ for binding sites (Sun et al. 2025), while K is crucial for maintaining cellular osmotic pressure and enzyme activity. Its preferential allocation to the shoot parts helps ensure photosynthetic and metabolic activity (Imtiaz et al. 2023). Mg is a core component of chlorophyll, and its reliable supply is crucial for maintaining photosynthetic capacity (Tränkner et al. 2018). Additionally, FM significantly increased plant uptake of Cu and Zn. Zn and Cd share similar chemical properties and uptake transport pathways, resulting in competitive relationships between them (Gupta et al. 2016; Song and Wang, 2017). The FM induced increase in plant Zn content may directly inhibit Cd uptake and upward transport by competing for absorption sites on root cell membranes or a classic ion antagonism protection mechanism. Concurrently, FM promotes the immobilization of Fe and Mn in roots. Fe/Mn oxides serve as crucial Cd adsorbents in soil, and the reduction and activation of Fe/Mn by roots and mycelium may be coupled with Cd fixation or release (Yang et al. 2023; Zeng et al. 2024). This FM mediated systemic selective uptake of mineral elements extends far beyond meeting basic nutritional requirements. It synergistically enhances Cd tolerance through at least three mechanisms: Firstly, nutritional enhancement: ensuring the supply of key elements such as P, K, and Mg to maintain basal metabolism and energy status, thereby improving overall plant health and stress resistance capacity; Secondly, competitive inhibition: Increasing uptake of competing ions like Zn directly reduces Cd entry; Thirdly, homeostasis and detoxification: stabilizing the cellular environment by regulating ion balances of Ca, Na potentially influencing Cd compartmentalization or chelation within cells.

## Conclusion

This study systematically elucidates the multi-interactive mechanisms by which FM enhances Cd tolerance in three *Sphagneticola* species (Fig. 6). Its action does not rely on a single pathway but begins with species-specific regulation of the rhizosphere pH microenvironment, subsequently acting on plants through a dual-pronged strategy: on one hand, by expanding the absorption range and activating the rhizosphere organic P pool, it directly and efficiently enhances P nutrient uptake, fundamentally alleviating P starvation caused by Cd stress; on the other hand, it comprehensively modulates plants mineral nutrient uptake and allocation patterns. By promoting the supply of antagonistic elements, signaling elements, and key nutrients, it constructs a multidimensional network against Cd toxicity across multiple levels including uptake sites, transport pathways, and intracellular homeostasis. Although the three *Sphagneticola* species exhibited differences in their specific responses to FM, reflecting the modulatory effects of their genetic backgrounds (native, invasive, and hybrid) on symbiotic functions, the positive effects of FM in promoting P uptake, activating rhizosphere ACP, and optimizing element profiles were consistently observed across all species. This confirms that the plant FM symbiotic system holds broad potential for enhancing tolerance, while also underscoring the importance of selecting optimal FM strain pairings for specific plant species. FM significantly enhances the Cd tolerance of *Sphagneticola* through the aforementioned synergistic mechanism. This not only provides new insights into the ecological function of mycorrhizal symbionts under stress conditions but also offers a solid theoretical basis and specific germplasm resource selection references for the practical application of combining *Sphagneticola* species with FM for phytostabilization or assisted phytoremediation of Cd contaminated soils.

## Data Availability

All data has been provided with the manuscript.

## Abbreviations

ACP: Acid phosphatase
ALP: Alkaline phosphatase
Cd: Cadmium
AMF: Arbuscular mycorrhizal fungi
FM: Funneliformis mosseae
